# Approaches to the evaluation of outbreak detection methods

**DOI:** 10.1186/1471-2458-6-263

**Published:** 2006-10-24

**Authors:** Rochelle E Watkins, Serryn Eagleson, Robert G Hall, Lynne Dailey, Aileen J Plant

**Affiliations:** 1 Australian Biosecurity CRC for Emerging Infectious Disease, Division of Health Sciences, Curtin University of Technology, Perth, Australia; 2 Department of Spatial Sciences, Curtin University of Technology, Perth, Australia; 3 Department of Human Services, Victoria, Australia

## Abstract

**Background:**

An increasing number of methods are being developed for the early detection of infectious disease outbreaks which could be naturally occurring or as a result of bioterrorism; however, no standardised framework for examining the usefulness of various outbreak detection methods exists. To promote comparability between studies, it is essential that standardised methods are developed for the evaluation of outbreak detection methods.

**Methods:**

This analysis aims to review approaches used to evaluate outbreak detection methods and provide a conceptual framework upon which recommendations for standardised evaluation methods can be based. We reviewed the recently published literature for reports which evaluated methods for the detection of infectious disease outbreaks in public health surveillance data. Evaluation methods identified in the recent literature were categorised according to the presence of common features to provide a conceptual basis within which to understand current approaches to evaluation.

**Results:**

There was considerable variation in the approaches used for the evaluation of methods for the detection of outbreaks in public health surveillance data, and appeared to be no single approach of choice. Four main approaches were used to evaluate performance, and these were labelled the Descriptive, Derived, Epidemiological and Simulation approaches. Based on the approaches identified, we propose a basic framework for evaluation and recommend the use of multiple approaches to evaluation to enable a comprehensive and contextualised description of outbreak detection performance.

**Conclusion:**

The varied nature of performance evaluation demonstrated in this review supports the need for further development of evaluation methods to improve comparability between studies. Our findings indicate that no single approach can fulfil all evaluation requirements. We propose that the cornerstone approaches to evaluation identified provide key contributions to support internal and external validity and comparability of study findings, and suggest these be incorporated into future recommendations for performance assessment.

## Background

The use of automated methods in public health surveillance for the early detection of naturally occurring or bioterrorism-related outbreaks aims to reduce the time between when an outbreak starts and when it is detected, allowing additional time for investigation and intervention for disease control. An increasing number of methods are being developed to detect outbreaks of infectious disease using routinely collected data, however, which automated surveillance method is best for detecting outbreaks is not easily determined.

A fundamental difficulty in the evaluation of outbreak detection methods involves specification of the aberration of interest [1]. Data aberrations, or changes in the distribution or frequency of important health-related events when compared with historical data, are not necessarily caused by an infectious disease outbreak, and may or may not be of public health importance [2]. In practice, determining a true increase in disease is problematic, often requiring considerable epidemiological judgement, which is complicated by its basis on a non-standard definition. Measurement of the validity of an outbreak detection method requires an operational definition of an outbreak [3], however, outbreaks are difficult to define precisely [4]. This creates challenges in determining appropriate criteria for examining the usefulness of outbreak detection methods.

The lack of standardised methods for the assessment of usefulness, including outbreak detection successes and failures, as well as the diversity of factors that influence performance, makes the comparison of methods and accumulation of knowledge in this area problematic [3]. This lack of comparability has consequences for knowledge development in the field, which affects both the developers of outbreak detection methods, as well as consumers of published research who are seeking to identify detection methods that may be most appropriate for a specific monitoring application. Well-developed evaluation and selection processes are required to determine the usefulness of outbreak detection methods. The performance of specific outbreak detection methods may be influenced by the evaluation approach used, thus consideration of the strengths and limitations of the evaluation approach used is essential.

There is a need for a standardised evaluation approach to allow the identification of methods which most successfully identify outbreaks under different conditions. Reviews published to date have examined whether outbreak detection methods have been evaluated, and which aspects have been evaluated [5,6], but none have examined in detail how these methods have been evaluated, nor provided any conceptual framework as a basis for further developments in the field. We identify the approaches used to evaluate the performance of automated outbreak detection methods, identify their major features, and place these within a broad conceptual framework in order to promote a better understanding of current approaches to the evaluation of outbreak detection methods.

## Methods

We reviewed reports in the recently published literature which document the evaluation of outbreak detection methods. We searched the Entrez PubMed electronic database using various combinations of the following search terms (surveillance, evaluate/evaluation, outbreak, epidemic, early detection, outbreak definition, epidemic definition, sensitivity, predictive value, automated, electronic) in September 2004 to identify relevant papers published since 1999. Several additional relevant papers were also obtained from a review of the reference lists of the publications retrieved. A second search was performed in October 2004 using the Web of Science search engine to locate papers published between 1999 and October 2004 that cited one of 18 key references (Table 1) identified from the original search. These key references were selected on the basis of their relevance to the review, their frequent citation in the relevant literature, and their use of a variety of outbreak detection methods.

Following the completion of the above searches, a Morbidity and Mortality Weekly Report Supplement (Volume 53) was published which included reports from a national syndromic surveillance conference. This volume was also reviewed for relevant papers.

The titles, abstracts and where appropriate the full text version of located publications were examined to determine inclusion in this review. Papers were excluded if they did not evaluate automated methods for the detection of outbreaks of infectious disease, did not provide information on the evaluation of outbreak detection methods described, included limited detail on the fields of interest (e.g. letters), were based on non-human data, were not published in English, contained data and evaluation methods very similar to those in a paper already reviewed, or presented forecasting or other statistical methods which were not evaluated in the context of the detection of outbreaks or bioterrorism-related events.

Papers documenting the evaluation of methods for the detection of outbreaks were reviewed, and characteristics of the evaluation approach used were recorded in an electronic database. Information recorded included the purpose of the surveillance, type of surveillance data analysed, data source, evaluation design (retrospective/prospective), and evaluation methods including the use of a criterion, criterion description, and whether different detection methods were compared. This abstracted information was reviewed and evaluation methods were classified according to the type of approach used. The classification developed is described and discussed.

The adequacy and comprehensiveness of the framework developed was subsequently investigated by assessing its ability to describe the methods used to evaluate outbreak detection methods reported in the recently-published literature. The Pubmed database was again searched for relevant papers published between January 2005 and July 2006, and the same inclusion criteria and review methods that were used for the original search were applied.

## Results

A total of 1418 unique references were obtained from the original PubMed search using 14 combinations of the selected search terms. Searches based on citations of the 18 key references (Table 1) located an additional 212 unique references, of which 164 were published between 1999 and September 2004 and reviewed for inclusion in this study.

Following a preliminary review of the references obtained, a total of 67 papers were considered to be highly relevant to the current study and were reviewed in detail. Four of these papers provided a limited amount of information related to the evaluation of outbreak detection performance. These papers primarily described or evaluated the implementation of a system for the early detection of outbreaks, and indicated that evaluation was incomplete due to the absence of alarms, the ongoing nature of system development, or practical constraints. This review will focus on the remaining 63 papers (see Additional file 1) that described the evaluation of outbreak detection methods in more detail.

Among the papers reviewed 41% reported the evaluation of syndromic or bioterrorism-related surveillance methods, 32% surveillance methods for specific diseases, 6% nosocomial infection surveillance methods, and 21% primarily described new analytic methods suitable for syndromic or disease-specific surveillance and provided illustrative evaluations. Almost two thirds of syndromic or bioterrorism-related surveillance methods reviewed analysed emergency department data, and over half (55%) of the disease-specific surveillance methods analysed disease notification data.

### Classification framework

**Figure 1:**
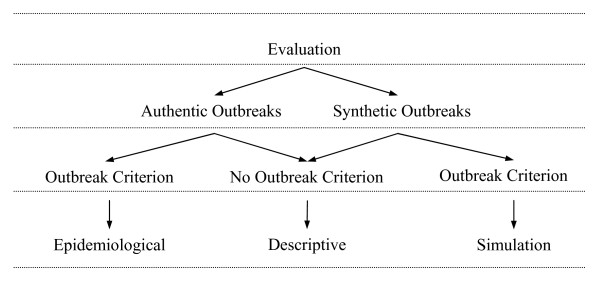
Framework for the classification of approaches used to evaluate outbreak detection methods for infectious disease surveillance.

As illustrated in the framework developed for classifying approaches to evaluation (Figure 1), papers reviewed used either authentic (73%) or synthetic (17%) outbreak data to evaluate performance, or both (10%). All disease-specific surveillance methods reviewed were evaluated using authentic data, with two studies (10%) also using synthetic data. Disease-specific surveillance methods were most likely to use a retrospective evaluation design (80%), with the remaining studies either reporting a prospective design (10%) or both retrospective and prospective designs (10%).

Syndromic or bioterrorism-related surveillance methods were also most commonly evaluated using authentic data (62%), with these studies being approximately equally likely to use a prospective or retrospective design. Of the 38% of syndromic or bioterrorism-related methods evaluated using synthetic data, 8 of these 10 studies used synthetic outbreak signals combined with authentic baseline data.

Approaches to evaluation can be further classified based on the use or non-use of a gold-standard criterion to define the occurrence of outbreaks, or events of interest (Figure 1). The majority (89%) of papers reviewed used some form of outbreak criterion to evaluate performance. Criterion-related methods allow the calculation of indicators of validity and timeliness with reference to the specific criterion used. Criteria selected often reflect commonly accepted or applied methods for determining outbreaks which may be disease or condition specific. For example, threshold-based methods were commonly used to indicate malaria outbreaks, and cyclic regression methods to indicate influenza outbreaks.

A number of papers that used criterion-related approaches to evaluation emphasised the difficulty involved in selecting a suitable criterion. Difficulties associated with the use of criterion-related approaches with authentic outbreak data include identifying the occurrence and exact timing of true outbreaks within the data, whereas difficulties associated with the use of criterion-related approaches with synthetic data include the specification of outbreak and baseline parameters to be used for evaluation. Retrospective evaluation using authentic data particularly highlights difficulties with the application of criterion-related methods, as available historical data may not consistently or comprehensively identify all outbreaks that occurred during the period of interest.

Methods used to evaluate outbreak detection performance can be further classified by the specific approach used to determine the occurrence of events of interest within the dataset. Four main methods were identified among the 63 papers reviewed, and these were labelled the Descriptive, Derived, Epidemiological and Simulation approaches. No single approach of choice was apparent among the papers reviewed, and 14% of studies used multiple approaches to evaluation. The prevalence and main features of the four specific approaches identified are summarised in Table 2, with studies categorised based on the predominant approaches used.

### 1. Descriptive approach

The Descriptive approach is characterised by the description of outbreak detection method performance, including the nature of events detected and the conditions under which alarms occur. The Descriptive approach differs from the other approaches identified in that indicators of performance are not based on a nominated outbreak criterion. This approach may be based on the assertion that it is not possible to accurately define the occurrence of all outbreaks in authentic data, or confirm changes detected as epidemiologically significant.

Descriptive indicators may incorporate qualitative and quantitative descriptors of both the data analysed and events detected, including incidence; seasonality; type of aberration detected; the frequency, timing and duration of alarms; the time between alarm and peak number of cases; and the magnitude of rise or proportion of cases before alarms. Although a limited amount of descriptive information on outbreak detection performance was often reported when other evaluation approaches were used, the Descriptive approach was the least commonly used approach in isolation, being most frequently applied in the early stages of performance evaluation.

The analysis by Hutwagner et al. [7] provides an example of the descriptive approach. The performance of five different detection algorithms based on four common historical datasets were described and compared without the use of a specific criterion. This analysis was able to highlight the different performance characteristics of the algorithms tested based on the two years of data analysed, including relative alert times, number of alerts generated, and differences in alerting patterns associated with characteristics such as disease frequency.

Descriptive evaluations can be difficult to compare between studies due to the large amount of information that may be relevant to the occurrence of outbreaks and their detection, and the often limited amount of data analysed. However, a specific strength of the Descriptive approach is that it can be effectively used to directly compare the performance of multiple outbreak detection methods using common data, where no single method is designated as the gold standard. Most (83%) of the studies reviewed that predominantly used a Descriptive approach compared different outbreak detection methods using common data.

A Descriptive approach can also be used to evaluate outbreak detection methods in relation to criteria other than outbreaks. Signals generated can be descriptively evaluated with reference to broad public health goals of surveillance based on existing understandings of disease and intervention capacity. For example, Teklehaimanot and co-workers [8] evaluated the potential impact of detection relative to potentially prevented cases, a concept which was based on a limited set of assumptions about the effectiveness of specific public health interventions.

### 2. Derived approach

The Derived approach is distinguished by the use of a standard indicator of outbreaks to derive performance measures from the data being analysed. Outbreak indicators are derived through the application of simple or complex data-derived models. The simplest examples of this approach involve the use of an absolute number of cases or statistically derived thresholds (for example based on standard deviations) to indicate the occurrence of an outbreak, which may be associated with a requirement to exceed a threshold for a minimum period of time. Complex models may incorporate multiple variables or methods to account for fluctuations in the surveillance data such as seasonal effects which result in varying outbreak criteria over time or space.

The study by Lewis et al [9] used the derived approach to investigate the effectiveness of different methods for the early detection of meningitis epidemics in Africa. Two main epidemic indicators were used to compare epidemic detection algorithms. These epidemic indicators were defined retrospectively based on the incidence rate of meningitis exceeding 70 and 100 cases per 100,000 inhabitants in one year. The epidemic peak was defined as the week with the highest incidence. Retrospective analysis of the data was then performed to compare the ability of a number of different subdistrict weekly incidence thresholds to detect the epidemics early. This method allows the timeliness of detection (relative to the epidemic peak), sensitivity, specificity and predictive value to be calculated for each detection method and compared. However, as identified by the authors, performance of the detection methods vary based on the gold standard criterion selected.

The Derived approach was most frequently used for the evaluation of disease-specific surveillance methods for the detection of large well-defined seasonal outbreaks. The models used reflect characteristics of the conditions under surveillance and the context of surveillance, for example, the rarity of the condition and the extent of background variability in the data. The evaluation of timeliness among studies using this approach was most commonly performed comparatively, based on the time of outbreak detection for several different detection methods, or the time to the epidemic peak. The Derived approach was typically associated with the use of a small number of variables to define the occurrence of an outbreak, which may provide a limited indicator of the occurrence of outbreaks within the data. For example, smaller outbreaks may be missed.

The definition of outbreaks used in the Derived approach combines elements of both the Descriptive and Epidemiological approaches as it is based on agreement with an alternative data model or algorithm which has some epidemiologic credibility. The Derived approach differs from the Descriptive approach in the specification of a gold standard criterion, and differs from the Epidemiological approach in the limited account of complexity considered in the specification of the criterion. Although this approach provides an operational definition of outbreaks, difficulties remain in the definition of properties of outbreaks, including the time of commencement. For these reasons the Derived approach is not considered entirely independent of the other approaches identified.

### 3. Epidemiological approach

This approach is most closely linked with traditional surveillance methods in the determination of the occurrence of an outbreak relative to some loosely-defined measure of expectation, and was the most commonly used approach to evaluation among the literature reviewed. Expert judgement is used to determine the occurrence of events of public health importance, often using traditional epidemiological investigation techniques. Expert judgement may be based on a variety of available information, including surveillance data and information from epidemiological investigations, and may vary in the extensiveness of investigation methods or data utilised to determine if a data aberration represents an outbreak. Typically judgements were based on multiple factors using flexible methods.

Terry and Huang's [10] analysis illustrates the use of the Epidemiological approach. Signals arising from a syndromic surveillance system were evaluated prospectively through epidemiological investigation of the events which produced the signal. The investigations followed a structured format, were conducted by an infectious-diseases physician and a non-physician epidemiologist, and involved the application of expert epidemiological opinion to determine if the signal was associated with an event of public health importance.

An advantage of the Epidemiological approach is that it allows complexities associated with the determination of occurrence of events of public health importance to be considered for each potential outbreak. However, epidemiological investigations can be resource intensive, and detailed descriptions of the investigations performed and the decision-making processes used are required to fully understand the basis of the outbreak definition applied. There is also evidence of variability in opinion among experts, and there has been little evaluation of the factors associated with this variability, or how it is best managed. Consensus among multiple raters has been used in a number of studies to control for individual variability.

A range of factors may influence expert opinion and decision-making relating to the occurrence of outbreaks, including specialist knowledge, previous experience and contextual information. Expert figures commonly used in the papers reviewed include epidemiologists, public health practitioners, public health physicians and infection control practitioners.

Approximately 40% of papers reviewed which used an Epidemiological approach used a prospective study design. Prospective surveillance of more than one data source can be used to promote a more comprehensive indicator of events of interest occurring by allowing the investigation of failures to signal as well as reasons for signalling. The use of official public health records or other published reports to identify known outbreaks was common among retrospective studies, and represents the application of traditional epidemiological methods for outbreak detection. Retrospective methods may suffer from incomplete ascertainment due to reliance on conventional methods and historical information, and inconsistencies in the methods used to identify outbreaks.

### 4. Simulation approach

Evaluation using a Simulation approach is based on criterion-related evaluation methods and requires that the definition of an outbreak be considered in the generation of data for evaluation. Studies that use synthetic data for evaluation using criterion-based methods are unique in that the number and timing of cases added to the baseline are known. Using synthetic data for evaluation addresses a number of problematic issues associated with the use of authentic data, including precisely determining the existence and timing of outbreaks within the data, and addressing a lack of data for evaluation and development. The Simulation approach is unique in enabling quantitative replicable evaluation of performance indicators including sensitivity and specificity with large sample sizes.

Reis and Mandl [11] used the Simulation approach to assess the performance of time series modelling for syndromic surveillance. The time series model performance was evaluated based on its ability to identify simulated outbreaks of different sizes. A total of 233 simulated outbreaks of 7 days in duration were inserted 15 days apart into a historical emergency department dataset that was free of known outbreaks. The simulation was repeated for outbreaks of different sizes, and the sensitivity of the time series models were compared at a fixed specificity.

Synthetic data can facilitate the comparison of multiple methods based on a standard dataset with specified outbreak and baseline characteristics. Approximately half of all studies which used synthetic data to evaluate performance used authentic baseline data with outbreak cases added. A comprehensive description of the simulated outbreaks and baseline data used in these evaluations is essential to allow their findings to be interpreted and integrated with those of other studies. The usefulness of synthetic data for evaluation is linked to the assumptions used to construct the data, which influences the ability to generalise evaluation findings to the authentic context. Both simple and complex outbreak simulation methods have been used to assess outbreak detection performance. Parameters that have been considered in the generation of synthetic data include outbreak size, outbreak shape, baseline rate and characteristics, and spatial distribution. Simulation methods also have the potential to influence the evaluation outcomes via effects produced by the simulation process which may not reflect the system or process being modelled.

Studies that used a Simulation approach for evaluation predominantly described the evaluation of syndromic surveillance methods or proposed new analysis methods for outbreak detection. This reflects the lack of authentic data available for evaluation of syndromic surveillance methods and the ability of synthetic datasets to allow comprehensive description of the performance of outbreak detection methods across a variety of scenarios.

### Recent trends

Our search of the literature published since 2005 located a total of 42 papers that were considered to be highly relevant to the current study. These papers were reviewed in detail to investigate the adequacy of the conceptual framework developed and describe current trends in the evaluation of outbreak detection methods.

The evaluation methods used by all papers reviewed were able to be described by the conceptual framework developed. The 42 studies located included 9 studies (21%) which were primarily descriptions or evaluations of new analysis methods that were not specific to outbreak detection or infectious disease surveillance, but were suitable for use in syndromic or disease-specific surveillance systems. Seven of these 9 studies described purely spatial analysis techniques. Of these 9 studies, 8 (89%) used a simulation approach to evaluate the performance of the algorithms, and 4 (44%) used a descriptive-comparative approach to illustrate and compare algorithm performance based on authentic data. Three studies used both a simulation and a descriptive-comparative approach to evaluation.

The remaining 33 papers described specific studies of surveillance systems and outbreak detection methods, and the approaches to evaluation used are summarised in Table 2. Among the papers reviewed 97% reported the evaluation of syndromic or bioterrorism-related surveillance methods. Thirty two studies (97%) used either epidemiological or simulation approaches to evaluation, with these approaches being approximately equally represented among the literature reviewed.

## Discussion

The primary goal of evaluating outbreak detection methods is to make inferences about their effectiveness. An unbiased assessment of performance is critical for identifying the most appropriate methods to use in specific monitoring applications. However, conclusions reached can be dependent upon the specific evaluation methods used, and consideration of the design of evaluation studies is essential in the interpretation of study findings.

Our review of a large sample of relevant published literature highlights the highly specific and varied nature of performance evaluation. Recent guidelines have been drafted for evaluating outbreak detection systems generally; however there are not yet any guidelines specific to performance assessment. As a result, a variety of criteria have been used to assess outbreak detection performance, and the majority of studies in the area do not provide comprehensive assessment of performance of the methods tested. These factors introduce barriers to the accumulation of knowledge in the field, as well as the wider application of the research to practice.

We describe a simple framework for the classification of approaches to the evaluation of outbreak detection methods. This framework identifies four specific approaches which are applied in the reviewed literature, and provides a logical structure within which to understand methods currently used for evaluation. The framework developed was found to be sufficient to describe the approaches used to evaluate outbreak detection methods in an independent sample of recently published studies. Based on the papers reviewed there does not appear to be any single approach of choice for the evaluation of methods for outbreak detection in public health surveillance data. A number of studies used multiple approaches to evaluation, indicating that any one approach may not satisfy all evaluation requirements, and highlighting the complementary nature of the approaches identified. The review of studies published since 2005 suggests the criterion-based simulation and epidemiological approaches to evaluation are the current approaches of choice, with the simulation approach becoming more commonly used. The recent development of tools which help to identify and simplify the technical demands of creating simulated data for evaluation [12-15] may promote more widespread use of simulation methods.

Multiple approaches to evaluation, including the use of authentic and synthetic data, allow the exploration of both applied and theoretical aspects of outbreak detection performance. Synthetic data are considered to allow more comprehensive characterisation of detection properties [16] and provide the most valid information for comparison of the different aberration detection methods [7], as they allow the manipulation of outbreak and baseline characteristics to cover a range of plausible scenarios, and the assessment of a large sample of outbreaks [17]. Synthetic data also allow an exact assessment of timeliness in relation to the first case. However, synthetic data are currently limited in their ability to mimic the diversity and unpredictability of actual outbreaks [3] and are associated with the risk of bias through evaluation under unrealistic conditions [18]. Evaluations using a simulation approach are more likely to have a greater level of internal validity than external validity, as the ability to generalise the findings of the study is dependent on the assumptions used to construct the data, and the influence of these assumptions on performance must be examined.

As recently highlighted by Sokolow et al. [20], authentic data provide an opportunity to test methods on the data upon which they will ultimately operate, and allow evaluation of the impact of unforseen influences on performance [19]. Studies that use authentic data can provide good support for the external validity of performance evaluations given sufficient replications are available for analysis [17]. However, a number of papers reviewed emphasised the difficulties associated with evaluating outbreak detection performance using authentic data, as there is no well-accepted gold standard which can be used to comprehensively define the occurrence of true outbreaks. The impact of uncertainty about the exact start and size of outbreaks on performance evaluations has also been highlighted by others [21,22]. Furthermore, the extent of evaluation in many studies which used authentic data was also limited by the infrequent occurrence of events of interest.

Approaches to defining outbreaks in authentic data for use in evaluation appear to vary according to the specific purpose and context of surveillance. The range of applied definitions of outbreaks reflect both practical constraints including the availability of sufficient data for evaluation, as well as the range of factors relevant to the determination of whether an outbreak has occurred for different surveillance purposes. For example, the specific methods used to distinguish outbreaks from background variation may include consideration of variables associated with potential causative factors, which may not be able to be specified in advance.

For public health surveillance purposes, the adequate definition of outbreaks is often problematic in the absence of sufficient epidemiological knowledge. This requirement for epidemiological knowledge is linked to the frequent use of an Epidemiological approach to evaluation, which allows consideration of complexity and causation in the evaluation of outbreak detection performance. Epidemiological indicators of outbreaks are not absolute due to their reliance on individual judgement; however, they provide the closest approximation to current practice, and are able to accommodate changing standards, expectations, response capacities, interventions and contextual factors more readily than methods using purely data-derived models. Furthermore, the use of prospective methods for the comprehensive investigation of alarms following their occurrence to determine their public health significance as well as the investigation of detection failures has specific advantages over retrospective methods. Retrospective methods do not allow evaluation of the extra sensitivity or specificity of outbreak detection methods, as signals from historical data which have not been detected by conventional means are classified as false positives [23].

Rare or highly variable events pose a specific challenge for evaluation. Although not commonly used among the studies reviewed, sensitivity analyses can be used for criterion-related approaches to address consequences of uncertainty or variation in detection goals. Criterion-related approaches have advantages over descriptive methods when the detection goal can be adequately defined, however their validity is dependent upon the assumptions used to construct outbreaks, as well as the comprehensiveness of the evaluation. The Descriptive approach provides an alternative approach to evaluation, particularly when there is no adequate definition of events of interest within a dataset, or when comparing outbreak detection methods.

The advantage of the Descriptive approach lies in the potential for systematic description of the key features of the aberrations detected and the data examined, and the comparison of multiple detection methods using common data. As the validity of outbreak detection methods may vary according to the outbreak scenario as well as surveillance system factors, different methods need to be evaluated under the same conditions to determine their relative value [3]. Although wider use of common test datasets would improve comparability between studies, given the broad range of applications, detection goals and contexts studied, the potential contribution of methods such as this is likely to be limited. The use of a descriptive approach to compare multiple methods within the surveillance context of interest using the same data may be a more feasible strategy, where the performance of suitable standard techniques could be reported to provide a basis for comparison.

The Descriptive approach requires further development to facilitate comparisons between outbreak detection methods through promoting more standardised, systematic and comprehensive descriptions of basic dataset features and measures of performance. Due to the potentially large reporting burden, further work is required to identify the attributes which would be most useful in standardised descriptions [6]. However, as illustrated in Figure 1, the Descriptive approach has the potential to promote an improved level of comparability between studies based on authentic and synthetic data.

Our review provides information on type and prevalence of approaches currently used to assess outbreak detection performance, and their strengths and limitations. We propose a basic framework to represent approaches currently used which provides a foundation for promoting increased comparability among studies and synthesis of knowledge in the field. This framework should offer assistance for both developers and consumers of outbreak detection research. Although there was considerable heterogeneity of study design within the approaches identified in this review, the type of approach used provides a reasonable guide to the strengths and limitations most relevant to specific studies.

None of the approaches identified is alone sufficient to provide a comprehensive assessment of outbreak detection performance. In light of the complementary nature of their strengths and limitations, the use of multiple approaches to evaluation where possible is recommended, as has been highlighted previously [17]. Although all evaluation approaches are not relevant to all research, for example some investigations may relate to an as yet hypothetical detection scenario, combined approaches can offer improved identification of comparative performance abilities, and more reliable estimates of performance under different conditions.

A major finding of this review is the identification of three of the four approaches described as 'cornerstone' approaches to evaluation, as they each use specific methods to address major requirements of the evaluation process. The key requirements of outbreak detection performance evaluations can be characterised by three main properties, being internal validity, external validity and comparability. These requirements can be related to the corresponding strengths of the three cornerstone approaches identified, being the Simulation, Epidemiological and Descriptive approaches respectively. As such, the use of multiple approaches to evaluation can provide the basis for a comprehensive and contextualised assessment of outbreak detection performance.

## Conclusion

Evaluation of the performance characteristics of outbreak detection methods is essential to allow an understanding of the type of outbreaks that can be identified, and how early these outbreaks can be identified [24]. The current lack of a standardised evaluation approach makes comparisons of the performance of different outbreak detection methods difficult. This review aimed to provide an inclusive description of approaches currently used to evaluate outbreak detection performance, leading to a clearer understanding of how outbreak detection methods are evaluated and the relative advantages and limitations of difference approaches.

Our findings indicate that no single approach can fulfil all evaluation requirements. The varied nature of performance evaluation demonstrated in this review supports the need for further development of evaluation methods as has been identified previously [3,6,25], to promote progress toward the development of more standardised methods. We propose that the three 'cornerstone' approaches to evaluation, the Simulation, Epidemiological, and Descriptive, approaches provide key contributions to the assessment of outbreak detection methods, supporting internal and external validity and comparability of study findings, and suggest these elements be incorporated into future recommendations for performance assessment.

## Competing interests

The author(s) declare that they have no competing interests.

## Authors' contributions

AJP and REW conceived and designed the study, REW conducted the literature review and content analysis and, REW, SE, RGH, LD and AJP were involved in finalizing the results, and drafting and critically revising the manuscript. All authors read and approved the final manuscript.

## Pre-publication history

The pre-publication history for this paper can be accessed here:



## Supplementary Material

Additional File 1 This file includes a list of all studies reviewed. Click here for file

## Figures and Tables

**Table 1:** Key papers used to conduct the Web of Science search 1999–2004 and search results

First author	Year	Source	Number of papers citing*
Serfling RE	1963	Public Health Rep; 78:494-506	48
Stroup DF	1989	Stat Med; 8:323-9	28
Kulldorff M	1995	Stat Med; 14:799-810	74
Farrington P	1996	J R Stat Soc A; 159:547-63	12
Hutwagner L	1997	Emerging Infect Dis; 3:395-400	28
Stern L	1999	Epidemiol Infect; 122:103-10	15
Kulldorff M	2001	J R Stat Soc A;164:61-72	16
Lazarus R	2001	BMC Public Health; 1:9	16
Rogerson PA	2001	J R Stat Soc A;164:87-96	11
Tsui FC	2001	Proc AMIA Symp; 2001:711-15	10
Wagner MM	2001	J Public Health Manag Pract;7:51-9	10
Barthell EN	2002	Ann Emerg Med; 39:422-9	13
Lober WB	2002	JAMIA; 9:105-15	23
Das D	2003	J Urban Health; 80:i76-88	7
Mostashari F	2003	Emerging Infect Dis; 9:641-6	9
Reis BY	2003	BMC Med Inform Decis Mak; 3(1):2	8
Sosin DM	2003	J Urban Health; 80:i8-13	5
Buehler JW	2004	MMWR Recomm Rep; 53:(RR-5)	2

* all years

**Table 2:** Approaches to evaluation identified

Approach	Main Feature	Main Limitation	1999–2004 Prevalence* n (%)	2005–2006 Prevalence** n (%)
Simulation	Outbreak signal specified	Generalisability of findings to the authentic context	17 (27%)	15 (45%)
Descriptive	No outbreak definition required	Evaluation not directly related to event of interest	12 (19%)	3 (9%)
Derived	Consistent outbreak definition used	Limited number of factors used in outbreak definition	18 (29%)	0 (0%)
Epidemiological	Complexity considered in outbreak definition	Potential variability, may be resource intensive	25 (40%)	17 (52%)

*9 of the 63 studies reviewed (14%) used multiple approaches to evaluation

**2 of the 33 studies reviewed (6%) used multiple approaches to evaluation
