# Supplementary material for: Approaches to the evaluation of outbreak detection methods
Source: BMC Public Health. 2006 Oct 24;6:263. doi: 10.1186/1471-2458-6-263 (PMC1626088; doi:10.1186/1471-2458-6-263)
Supplement: Additional File 1 — This file includes a list of all studies reviewed. [file 1471-2458-6-263-S1.pdf]

Annex

Review 1

1. Baker M, Smith GE, Cooper D, Verlander NQ, Chinemana F, Cotterill S, Hollyoak V, Griffiths R: **Early warning and NHS Direct: a role in community surveillance?** *J Public Health Med* 2003, **25**(4):362-368.
2. Brown SM, Benneyan JC, Theobald DA, Sands K, Hahn MT, Potter-Bynoe GA, Stelling JM, O'Brien TF, Goldmann DA: **Binary cumulative sums and moving averages in nosocomial infection cluster detection**. *Emerg Infect Dis* 2002, **8**(12):1426-1432.
3. Buckeridge DL, Graham J, O'Connor MJ, Choy MK, Tu SW, Musen MA: **Knowledge-based bioterrorism surveillance**. *Proc AMIA Symp* 2002:76-80.
4. Burkom HS: **Biosurveillance applying scan statistics with multiple, disparate data sources**. *J Urban Health* 2003, **80**(2 Suppl 1):i57-65.
5. Carme B, Sobesky M, Biard MH, Cotellon P, Aznar C, Fontanella JM: **Non-specific alert system for dengue epidemic outbreaks in areas of endemic malaria. A hospital-based evaluation in Cayenne (French Guiana)**. *Epidemiol Infect* 2003, **130**(1):93-100.
6. Dafni UG, Tsiodras S, Panagiotakos D, Gkolfinopoulou K, Kouvatseas G, Tsourti Z, Saroglou G: **Algorithm for statistical detection of peaks--syndromic surveillance system for the Athens 2004 Olympic Games**. *MMWR Morb Mortal Wkly Rep* 2004, **53 Suppl**:86-94.
7. Das D, Weiss D, Mostashari F, Treadwell T, McQuiston J, Hutwagner L, Karpati A, Bornschlegel K, Seeman M, Turcios R *et al*: **Enhanced drop-in syndromic surveillance in New York City following September 11, 2001**. *J Urban Health* 2003, **80**(2 Suppl 1):i76-88.
8. de Chabalier F, Djingarey MH, Hassane A, Chippaux JP: **Meningitis seasonal pattern in Africa and detection of epidemics: a retrospective study in Niger, 1990-98**. *Trans R Soc Trop Med Hyg* 2000, **94**(6):664-668.
9. de Chabalier F, Hassane A, Chippaux JP: **Evaluation of surveillance thresholds for prediction of meningitis epidemics using ongoing surveillance data at the district level, in Niger**. *Trans R Soc Trop Med Hyg* 2000, **94**(3):251-252.
10. Dembek ZF, Carley K, Siniscalchi A, Hadler J: **Hospital admissions syndromic surveillance--Connecticut, September 200-November 2003**. *MMWR Morb Mortal Wkly Rep* 2004, **53 Suppl**:50-52.
11. Farrington CP, Andrews NJ, Beale AD, Catchpole MA: **A statistical algorithm for the early detection of outbreaks of infectious disease**. *J R Stat Soc Ser A* 1996, **159**:547-563.
12. Goldenberg A, Shmueli G, Caruana RA, Fienberg SE: **Early statistical detection of anthrax outbreaks by tracking over-the-counter medication sales**. *Proc Natl Acad Sci U S A* 2002, **99**(8):5237-5240.
13. Gustafson TL: **Practical risk-adjusted quality control charts for infection control**. *Am J Infect Control* 2000, **28**(6):406-414.
14. Hacek DM, Cordell RL, Noskin GA, Peterson LR: **Computer-assisted surveillance for detecting clonal outbreaks of nosocomial infection**. *J Clin Microbiol* 2004, **42**(3):1170-1175.
15. Hashimoto S, Murakami Y, Taniguchi K, Nagai M: **Detection of epidemics in their early stage through infectious disease surveillance**. *Int J Epidemiol* 2000, **29**(5):905-910.
16. Hay SI, Simba M, Busolo M, Noor AM, Guyatt HL, Ochola SA, Snow RW: **Defining and detecting malaria epidemics in the highlands of western Kenya**. *Emerg Infect Dis* 2002, **8**(6):555-562.
17. Heffernan R, Mostashari F, Das D, Kulldorff M, Weiss D: **Syndromic surveillance in public health practice, New York City**. *Emerg Infect Dis* 2004, **10**(5):858-864.
18. Heino J, Toivonen H: **Automated detection of epidemics from the usage logs of a physicians' reference database**. In: *Knowledge Discovery in Databases: Pkdd 2003.* Vol. 2838; 2003: 180-191.
19. Hogan WR, Tsui FC, Ivanov O, Gesteland PH, Grannis S, Overhage JM, Robinson JM, Wagner MM: **Detection of pediatric respiratory and diarrheal outbreaks from sales of over-the-counter electrolyte products**. *J Am Med Inform Assoc* 2003, **10**(6):555-562.
20. Hutwagner LC, Maloney EK, Bean NH, Slutsker L, Martin SM: **Using laboratory-based surveillance data for prevention: An algorithm for detecting Salmonella outbreaks**. *Emerg Infect Dis* 1997, **3**(3):395-400.

21. Hutwagner L, Thompson W, Groseclose S, Williamson G: **An evaluation of alternative methods for detecting aberrations in public health surveillance data.** In: *American Statistical Association, Joint Statistical Meetings, Proceedings of the Biometrics Section: August 2000; Indianapolis*; 2000: 82–85.

22. Irvin CB, Nouhan PP, Rice K: **Syndromic analysis of computerized emergency department patients' chief complaints: an opportunity for bioterrorism and influenza surveillance**. *Ann Emerg Med* 2003, **41**(4):447-452.

23. Ivanov O, Gesteland PH, Hogan W, Mundorff MB, Wagner MM: **Detection of pediatric respiratory and gastrointestinal outbreaks from free-text chief complaints**. *AMIA Annu Symp Proc* 2003:318-322.

24. Kaninda AV, Belanger F, Lewis R, Batchassi E, Aplogan A, Yakoua Y, Paquet C: **Effectiveness of incidence thresholds for detection and control of meningococcal meningitis epidemics in northern Togo**. *Int J Epidemiol* 2000, **29**(5):933-940.

25. Kleinman K, Lazarus R, Platt R: **A generalized linear mixed models approach for detecting incident clusters of disease in small areas, with an application to biological terrorism**. *Am J Epidemiol* 2004, **159**(3):217-224.

26. Koch M, McKenna S, Bilisoly R: **Automatic Syndrome Surveillance Using Space-Time Clustering**. In: *MSS National Symposium on Sensor and Data Fusion: August 13-15 2002; San Diego, California*; 2002.

27. Kulldorff M, Zhang Z, Hartman J, Heffernan R, Huang L, Mostashari F: **Benchmark data and power calculations for evaluating disease outbreak detection methods**. *MMWR Morb Mortal Wkly Rep* 2004, **53 Suppl**:144-151.

28. Lazarus R, Kleinman K, Dashevsky I, Adams C, Kludt P, DeMaria A, Jr., Platt R: **Use of automated ambulatory-care encounter records for detection of acute illness clusters, including potential bioterrorism events**. *Emerg Infect Dis* 2002, **8**(8):753-760.

29. Le Strat Y, Carrat F: **Monitoring epidemiologic surveillance data using hidden Markov models**. *Stat Med* 1999, **18**(24):3463-3478.

30. Leake JA, Kone ML, Yada AA, Barry LF, Traore G, Ware A, Coulibaly T, Berthe A, Mambu Ma Disu H, Rosenstein NE *et al*: **Early detection and response to meningococcal disease epidemics in sub-Saharan Africa: appraisal of the WHO strategy**. *Bull World Health Organ* 2002, **80**(5):342-349.

31. Lewis R, Nathan N, Diarra L, Belanger F, Paquet C: **Timely detection of meningococcal meningitis epidemics in Africa**. *Lancet* 2001, **358**(9278):287-293.

32. Lewis MD, Pavlin JA, Mansfield JL, O'Brien S, Boomsma LG, Elbert Y, Kelley PW: **Disease outbreak detection system using syndromic data in the greater Washington DC area**. *Am J Prev Med* 2002, **23**(3):180-186.

33. Lombardo JS: **The ESSENCE II disease surveillance test bed for the national capital area**. *Johns Hopkins APL Tech Dig* 2003, **24**(4):327-334.

34. McKenna S, Bilisoly R, Koch M: **Comparing Techniques for Detection of Epidemics in Public Health Surveillance Data**. In: *BTR 2002: Unified Science and Technology for Reducing Biological Threats and Countering Terrorism: March 14-15 2002; University of New Mexico, Albuquerque, New Mexico*; 2002.

35. Molinari N, Bonaldi C, Daures JP: **Multiple temporal cluster detection**. *Biometrics* 2001, **57**(2):577-583.

36. Mostashari F, Fine A, Das D, Adams J, Layton M: **Use of ambulance dispatch data as an early warning system for communitywide influenzalike illness, New York City**. *J Urban Health* 2003, **80**(2 Suppl 1):i43-49.

37. Mostashari F, Kulldorff M, Hartman JJ, Miller JR, Kulasekera V: **Dead bird clusters as an early warning system for West Nile virus activity**. *Emerg Infect Dis* 2003, **9**(6):641-646.

38. Murakami Y, Hashimoto S, Taniguchi K, Osaka K, Fuchigami H, Nagai M: **Evaluation of a method for issuing warnings pre-epidemics and epidemics in Japan by infectious diseases surveillance**. *J Epidemiol* 2004, **14**(2):33-40.

39. Ozonoff A, Forsberg L, Bonetti M, Pagano M: **Bivariate method for spatio-temporal syndromic surveillance**. *MMWR Morb Mortal Wkly Rep* 2004, **53 Suppl**:61-66.

40. Rath TM, Carreras M, Sebastiani P: **Automated detection of influenza epidemics with Hidden Markov Models**. In: *Advances in Intelligent Data Analysis V.* vol. 2810; 2003: 521-532.

41. Reis BY, Mandl KD: **Time series modeling for syndromic surveillance**. *BMC Med Inform Decis Mak* 2003, **3**(1):2.
42. Reis BY, Mandl KD: **Integrating syndromic surveillance data across multiple locations: effects on outbreak detection performance**. *Proc AMIA Symp* 2003:549-553.
43. Reis BY, Pagano M, Mandl KD: **Using temporal context to improve biosurveillance**. *Proc Natl Acad Sci U S A* 2003, **100**(4):1961-1965.
44. Reis BY, Mandl KD: **Syndromic surveillance: the effects of syndrome grouping on model accuracy and outbreak detection**. *Ann Emerg Med* 2004, **44**(3):235-241.
45. Rigau-Perez JG, Millard PS, Walker DR, Deseda CC, Casta-Velez A: **A deviation bar chart for detecting dengue outbreaks in Puerto Rico**. *Am J Public Health* 1999, **89**(3):374-378.
46. Rogerson PA: **Monitoring point patterns for the development of space-time clusters**. *J R Stat Soc Ser A* 2001, **164**:87-96.
47. Rogerson PA, Yamada I: **Monitoring change in spatial patterns of disease: comparing univariate and multivariate cumulative sum approaches**. *Stat Med* 2004, **23**(14):2195-2214.
48. Rogerson PA, Yamada I: **Approaches to syndromic surveillance when data consist of small regional counts**. *MMWR Morb Mortal Wkly Rep* 2004, **53 Suppl**:79-85.
49. Rossi G, Lampugnani L, Marchi M: **An approximate CUSUM procedure for surveillance of health events**. *Stat Med* 1999, **18**(16):2111-2122.
50. Sauders BD, Fortes ED, Morse DL, Dumas N, Kiehlbauch JA, Schukken Y, Hibbs JR, Wiedmann M: **Molecular subtyping to detect human listeriosis clusters**. *Emerg Infect Dis* 2003, **9**(6):672-680.
51. Siegrist D, Pavlin J: **Bio-ALIRT biosurveillance detection algorithm evaluation**. *MMWR Morb Mortal Wkly Rep* 2004, **53 Suppl**:152-158.
52. Steiner-Sichel L, Greenko J, Heffernan R, Layton M, Weiss D: **Field investigations of emergency department syndromic surveillance signals--New York City**. *MMWR Morb Mortal Wkly Rep* 2004, **53 Suppl**:184-189.
53. Stern L, Lightfoot D: **Automated outbreak detection: a quantitative retrospective analysis**. *Epidemiol Infect* 1999, **122**(1):103-110.
54. Stroup DF, Williamson GD, Herndon JL, Karon JM: **Detection of aberrations in the occurrence of notifiable diseases surveillance data**. *Stat Med* 1989, **8**(3):323-329.
55. Teklehaimanot HD, Schwartz J, Teklehaimanot A, Lipsitch M: **Alert threshold algorithms and malaria epidemic detection**. *Emerg Infect Dis* 2004, **10**(7):1220-1226.
56. Terry W, Ostrowsky B, Huang A: **Should we be worried? Investigation of signals generated by an electronic syndromic surveillance system-Westchester County, New York**. *MMWR Morb Mortal Wkly Rep* 2004, **53 (Suppl)**:190-195.
57. Theophilides CN, Ahearn SC, Grady S, Merlino M: **Identifying West Nile virus risk areas: The dynamic continuous-area space-time system**. *Am J Epidemiol* 2003, **157**(9):843-854.
58. Tsui FC, Wagner MM, Dato V, Chang CC: **Value of ICD-9 coded chief complaints for detection of epidemics**. *J Am Med Inf Assoc* 2002, **9**(6):S41-S47.
59. Weber SG, Pitrak D: **Accuracy of a local surveillance system for early detection of emerging infectious disease**. *Jama* 2003, **290**(5):596-598.
60. Williamson GD, Hudson GW: **A monitoring system for detecting aberrations in public health surveillance reports**. *Stat Med* 1999, **18**(23):3283-3298.
61. Wong WK, Moore A, Cooper G, Wagner M: **WSARE: What's Strange About Recent Events?** *J Urban Health* 2003, **80**(2 Suppl 1):i66-75.
62. Wright MO, Perencevich EN, Novak C, Hebden JN, Standiford HC, Harris AD: **Preliminary assessment of an automated surveillance system for infection control**. *Infect Control Hosp Epidemiol* 2004, **25**(4):325-332.
63. Yuan CM, Love S, Wilson M: **Syndromic surveillance at hospital emergency departments--southeastern Virginia**. *MMWR Morb Mortal Wkly Rep* 2004, **53 Suppl**:56-58.

Review 2

1. Aamodt G, Samuelsen SO, Skrondal A: **A simulation study of three methods for detecting disease clusters**. *Int J Health Geogr* 2006, **5**:15.
2. Ang BC, Chen MI, Goh TL, Ng YY, Fan SW: **An assessment of electronically captured data in the patient care enhancement system (PACES) for syndromic surveillance**. *Ann Acad Med Singapore* 2005, **34**(9):539-534.
3. Assuncao R, Costa M, Tavares A, Ferreira S: **Fast detection of arbitrarily shaped disease clusters**. *Stat Med* 2006, **25**(5):723-742.
4. Balter S, Weiss D, Hanson H, Reddy V, Das D, Heffernan R: **Three years of emergency department gastrointestinal syndromic surveillance in New York City: what have we found?** *MMWR Morb Mortal Wkly Rep* 2005, **54 Suppl**:175-180.
5. Besculides M, Heffernan R, Mostashari F, Weiss D: **Evaluation of school absenteeism data for early outbreak detection, New York City**. *BMC Public Health* 2005, **5**:105.
6. Bonetti M, Pagano M: **The interpoint distance distribution as a descriptor of point patterns, with an application to spatial disease clustering**. *Stat Med* 2005, **24**(5):753-773.
7. Bourgeois FT, Olson KL, Brownstein JS, McAdam AJ, Mandl KD: **Validation of syndromic surveillance for respiratory infections**. *Ann Emerg Med* 2006, **47**(3):265 e261.
8. Buckeridge DL, Switzer P, Owens D, Siegrist D, Pavlin J, Musen M: **An evaluation model for syndromic surveillance: assessing the performance of a temporal algorithm**. *MMWR Morb Mortal Wkly Rep* 2005, **54 Suppl**:109-115.
9. Burkom HS, Murphy S, Coberly J, Hurt-Mullen K: **Public health monitoring tools for multiple data streams**. *MMWR Morb Mortal Wkly Rep* 2005, **54 Suppl**:55-62.
10. Cassa CA, Grannis SJ, Overhage JM, Mandl KD: **A context-sensitive approach to anonymizing spatial surveillance data: impact on outbreak detection**. *J Am Med Inform Assoc* 2006, **13**(2):160-165.
11. Chen MI, Tan IB, Ng YY: **Modelling the utility of body temperature readings from primary care consults for SARS surveillance in an army medical centre**. *Ann Acad Med Singapore* 2006, **35**(4):236-241.
12. Chen JH, Schmit K, Chang H, Herlihy E, Miller J, Smith P: **Use of Medicaid prescription data for syndromic surveillance--New York**. *MMWR Morb Mortal Wkly Rep* 2005, **54 Suppl**:31-34.
13. Cooper DL, Verlander NQ, Smith GE, Charlett A, Gerard E, Willocks L, O'Brien S: **Can syndromic surveillance data detect local outbreaks of communicable disease? A model using a historical cryptosporidiosis outbreak**. *Epidemiol Infect* 2006, **134**(1):13-20.
14. Das D, Metzger K, Heffernan R, Balter S, Weiss D, Mostashari F: **Monitoring over-the-counter medication sales for early detection of disease outbreaks--New York City**. *MMWR Morb Mortal Wkly Rep* 2005, **54 Suppl**:41-46.
15. Doroshenko A, Cooper D, Smith G, Gerard E, Chinemana F, Verlander N, Nicoll A: **Evaluation of syndromic surveillance based on National Health Service Direct derived data--England and Wales**. *MMWR Morb Mortal Wkly Rep* 2005, **54 Suppl**:117-122.
16. Duczmal L, Buckeridge DL: **A workflow spatial scan statistic**. *Stat Med* 2006, **25**(5):743-754.
17. Hadler JL, Siniscalchi A, Dembek Z: **Hospital admissions syndromic surveillance--Connecticut, October 2001-June 2004**. *MMWR Morb Mortal Wkly Rep* 2005, **54 Suppl**:169-173.
18. Hossain MM, Lawson AB: **Cluster detection diagnostics for small area health data: with reference to evaluation of local likelihood models**. *Stat Med* 2006, **25**(5):771-786.
19. Hutwagner L, Browne T, Seeman GM, Fleischauer AT: **Comparing aberration detection methods with simulated data**. *Emerg Infect Dis* 2005, **11**(2):314-316.
20. Hutwagner LC, Thompson WW, Seeman GM, Treadwell T: **A simulation model for assessing aberration detection methods used in public health surveillance for systems with limited baselines**. *Stat Med* 2005, **24**(4):543-550.
21. Iyengar VS: **Space-time clusters with flexible shapes**. *MMWR Morb Mortal Wkly Rep* 2005, **54 Suppl**:71-76.

22. Kleinman KP, Abrams A, Mandl K, Platt R: **Simulation for assessing statistical methods of biologic terrorism surveillance**. *MMWR Morb Mortal Wkly Rep* 2005, **54 Suppl**:101-108.
23. Kleinman KP, Abrams AM, Kulldorff M, Platt R: **A model-adjusted space-time scan statistic with an application to syndromic surveillance**. *Epidemiol Infect* 2005, **133**(3):409-419.
24. Kleinman K, Abrams A, Katherine Yih W, Platt R, Kulldorff M: **Evaluating spatial surveillance: detection of known outbreaks in real data**. *Stat Med* 2006, **25**(5):755-769.
25. Kulldorff M, Heffernan R, Hartman J, Assuncao R, Mostashari F: **A space-time permutation scan statistic for disease outbreak detection**. *PLoS Med* 2005, **2**(3):e59.
26. Lawson AB: **Disease cluster detection: a critique and a Bayesian proposal**. *Stat Med* 2006, **25**(5):897-916.
27. Louie MM, Kolaczyk ED: **Multiscale detection of localized anomalous structure in aggregate disease incidence data**. *Stat Med* 2006, **25**(5):787-810.
28. Mohtashemi M, Szolovits P, Dunyak J, Mandl KD: **A susceptible-infected model of early detection of respiratory infection outbreaks on a background of influenza**. *J Theor Biol* 2006, **241**(4):954-963.
29. Najmi AH, Magruder SF: **An adaptive prediction and detection algorithm for multistream syndromic surveillance**. *BMC Med Inform Decis Mak* 2005, **5**:33.
30. Naumova EN, O'Neil E, MacNeill I: **INFERNO: a system for early outbreak detection and signature forecasting**. *MMWR Morb Mortal Wkly Rep* 2005, **54 Suppl**:77-83.
31. Nordin JD, Goodman MJ, Kulldorff M, Ritzwoller DP, Abrams AM, Kleinman K, Levitt MJ, Donahue J, Platt R: **Simulated anthrax attacks and syndromic surveillance**. *Emerg Infect Dis* 2005, **11**(9):1394-1398.
32. Ohkusa Y, Shigematsu M, Taniguchi K, Okabe N: **Experimental surveillance using data on sales of over-the-counter medications--Japan, November 2003-April 2004**. *MMWR Morb Mortal Wkly Rep* 2005, **54 Suppl**:47-52.
33. Olson KL, Bonetti M, Pagano M, Mandl KD: **Real time spatial cluster detection using interpoint distances among precise patient locations**. *BMC Med Inform Decis Mak* 2005, **5**:19.
34. Ritzwoller DP, Kleinman K, Palen T, Abrams A, Kaferly J, Yih W, Platt R: **Comparison of syndromic surveillance and a sentinel provider system in detecting an influenza outbreak--Denver, Colorado, 2003**. *MMWR Morb Mortal Wkly Rep* 2005, **54 Suppl**:151-156.
35. Rolfhamre P, Ekdahl K: **An evaluation and comparison of three commonly used statistical models for automatic detection of outbreaks in epidemiological data of communicable diseases**. *Epidemiol Infect* 2006, **134**(4):863-871.
36. Rolland E, Moore KM, Robinson VA, McGuinness D: **Using Ontario's "Telehealth" health telephone helpline as an early-warning system: a study protocol**. *BMC Health Serv Res* 2006, **6**:10.
37. Sokolow LZ, Grady N, Rolka H, Walker D, McMurray P, English-Bullard R, Loonsk J: **Deciphering data anomalies in BioSense**. *MMWR Morb Mortal Wkly Rep* 2005, **54 Suppl**:133-139.
38. Takahashi K, Tango T: **An extended power of cluster detection tests**. *Stat Med* 2006, **25**(5):841-852.
39. Wallstrom GL, Wagner M, Hogan W: **High-fidelity injection detectability experiments: a tool for evaluating syndromic surveillance systems**. *MMWR Morb Mortal Wkly Rep* 2005, **54 Suppl**:85-91.
40. Wang L, Ramoni MF, Mandl KD, Sebastiani P: **Factors affecting automated syndromic surveillance**. *Artif Intell Med* 2005, **34**(3):269-278.
41. Yih KW, Abrams A, Danila R, Green K, Kleinman K, Kulldorff M, Miller B, Nordin J, Platt R: **Ambulatory-care diagnoses as potential indicators of outbreaks of gastrointestinal illness--Minnesota**. *MMWR Morb Mortal Wkly Rep* 2005, **54 Suppl**:157-162.
42. Zhu Y, Wang W, Atrubin D, Wu Y: **Initial evaluation of the early aberration reporting system--Florida**. *MMWR Morb Mortal Wkly Rep* 2005, **54 Suppl**:123-130.
